# Neoadjuvant chemoradiation compared to neoadjuvant radiation alone and surgery alone for Stage II and III soft tissue sarcoma of the extremities

**DOI:** 10.1186/1748-717X-6-91

**Published:** 2011-08-09

**Authors:** Kelly K Curtis, Jonathan B Ashman, Christopher P Beauchamp, Adam J Schwartz, Matthew D Callister, Amylou C Dueck, Leonard L Gunderson, Tom R Fitch

**Affiliations:** 1 Department of Internal Medicine, Division of Hematology/Oncology, Mayo Clinic, 13400 East Shea Blvd., Scottsdale, AZ 85259, USA; 2 Department of Radiation Oncology, Mayo Clinic, 13400 East Shea Blvd., Scottsdale, AZ 85259, USA; 3 Department of Surgery, Division of Orthopedic Surgery, Mayo Clinic, 5779 East Mayo Blvd., Phoenix, AZ 85054, USA; 4 Division of Biomedical Statistics and Informatics, Mayo Clinic, 13400 East Shea Blvd., Scottsdale, AZ 85259, USA

**Keywords:** Neoadjuvant, chemotherapy, radiation, chemoradiation, soft tissue sarcoma, extremity

## Abstract

**Background:**

Neoadjuvant chemoradiation (NCR) prior to resection of extremity soft tissue sarcoma (STS) has been studied, but data are limited. We present outcomes with NCR using a variety of chemotherapy regimens compared to neoadjuvant radiation without chemotherapy (NR) and surgery alone (SA).

**Methods:**

We conducted a retrospective chart review of 112 cases.

**Results:**

Treatments included SA (36 patients), NCR (39 patients), and NR (37 patients). NCR did not improve the rate of margin-negative resections over SA or NR. Loco-regional relapse-free survival, distant metastases-free survival, and overall survival (OS) were not different among the treatment groups. Patients with relapsed disease (OR 11.6; p = 0.01), and tumor size greater than 5 cm (OR 9.4; p = 0.01) were more likely to have a loco-regional recurrence on logistic regression analysis. Significantly increased OS was found among NCR-treated patients with tumors greater than 5 cm compared to SA (3 year OS 69 vs. 40%; p = 0.03). Wound complication rates were higher after NCR compared to SA (50 vs. 11%; p = 0.003) but not compared to NR (p = 0.36). Wet desquamation was the most common adverse event of NCR.

**Conclusions:**

NCR and NR are acceptable strategies for patients with STS. NCR is well-tolerated, but not clearly superior to NR.

## Background

Extremity soft tissue sarcoma (STS) treatment strategies gradually have shifted away from amputation toward a limb preservation approach. For most patients with low-grade extremity STS, (i.e., T1-2, N0, M0) surgical resection is the primary treatment, followed by adjuvant radiation for margins less than or equal to 1 centimeter [1]. For patients with high-grade STS of the extremities (i.e., Stages II or III), neoadjuvant radiation with or without chemotherapy often is employed to improve local control and functional outcome [1].

Experience with neoadjuvant chemoradiation (NCR) in STS has been reported by several groups. Eilber and colleagues published a regimen of intra-arterial doxorubicin infused over 24-hours for 3 days prior to radiation, followed by surgery [2]. Other single agents that have been studied with pre-operative radiation include ifosfamide and gemcitabine [3,4]. Multi-agent chemotherapy regimens given pre-operatively with radiation include MAID (mesna, doxorubicin, ifosfamide and dacarbazine) or IMAP/MAP (ifosfamide, mitomycin, doxorubicin, and cisplatin) [5-7]. These strategies have shown promising results, including 5-year overall survival rates up to 70% [8-11], 5-year local control rates up to 92% [5] and limb preservation rates up to 100% [4]. Toxicities of NCR typically include wound complications, many of which require re-operation, and long bone fracture [12].

At Mayo Clinic in Arizona (MCA), the decision to use NCR, neoadjuvant radiation (NR) or surgery alone (SA) is based on initial magnetic resonance imaging (MRI) findings. Patients likely to have narrow resection margins, with high grade tumors, large tumor size, and an unfavorable location relative to the neuro-vascular bundles and bone are referred to radiation oncology and medical oncology for consideration of NR or NCR. Despite its use, data on outcomes with NCR for Stage II and III extremity STS are limited. A prospective, randomized trial comparing NCR to NR and SA is needed to provide more robust knowledge. In the absence of such information, a retrospective analysis can provide preliminary insight and be used for hypothesis generation. Therefore, we conducted a retrospective analysis of patients with extremity STS treated at MCA to increase our understanding of NCR-related outcomes as compared to NR- and SA-treated patients.

## Methods

A retrospective chart review was conducted of 112 extremity STS cases treated between January 1, 1998 and December 31, 2009 at MCA. We included patients greater than 15 years of age with Stage II and III extremity STS as defined by the 2010 7^th ^Edition American Joint Committee on Cancer (AJCC) Staging System of STS. Patients with relapsed extremity STS being treated with curative intent were included. Non-extremity sarcomas, low grade (Stage I) extremity STS, and bone/cartilage sarcomas were excluded. Patients treated with post-operative radiation and patients with metastatic or recurrent disease receiving only palliative treatments were excluded. The review was approved by the Mayo Clinic Institutional Review Board.

The following information was recorded: age at diagnosis, date of first MCA evaluation, sex, primary disease site, histology, grade, tumor size and depth (superficial or deep as defined by the 2010 AJCC Staging System of STS), margin status, notation of periosteal or nerve stripping in the operative summary, limb preservation or amputation, occurrence of wound complications following surgery, date of first local recurrence (if any), date of appearance of distant metastases (if any), any documentation of treatment-related toxicity, and date of death or last follow-up at MCA. It was not possible to determine toxicity grading from medical records. Sarcoma treatment was categorized as follows: SA (defined as any curative-intent surgical procedure performed without pre- or post-operative chemotherapy or radiation), NCR (defined as any combination of chemotherapy with radiation given prior to a curative-intent surgical resection), or NR (defined as radiation given without chemotherapy prior to a curative-intent surgical resection). Patients treated with sequential pre-operative chemotherapy followed by pre-operative radiation were included in the NCR group, since historically such therapy has been considered a form of NCR [2,13]. Use of intra-operative electron radiation therapy (IOERT) or perioperative brachytherapy was documented.

Surgical margins were recorded as negative (R0 resection) if the pathology report noted all margins to be free of tumor microscopically. If tumor extended to the surgical margin microscopically, or if the surgical margin was less than or equal to 1 mm, the margin was considered to be positive (R1 resection). It was not possible to determine pathologic response rates to NCR or NR from the records. Loco-regional recurrences were defined as any relapse of sarcoma at the previous surgical site or in regional lymph nodes. A "wound complication" was defined as any post-operative wound event requiring a return to the operating room for an unplanned additional procedure.

All time-to-failure endpoints were calculated from the date of first MCA contact. Overall survival (OS) was defined as death as a result of any cause; time to loco-regional recurrence was defined as time to date of a local or regional relapse diagnosis or amputation for any reason; time to distant metastases was defined as time to date of discovery of distant metastases, excluding new primary cancers. Kaplan-Meier methods were used to estimate OS, loco-regional relapse-free survival (LR-RFS), and distant metastasis-free survival (DMFS) for each of the treatment modality received. Contingency analyses using the Chi-square test of independence were conducted for different treatment modalities and surgical outcome, limb preservation, presence or absence of local recurrence and distant metastases, and presence or absence of wound complications. Logistic regression analyses were performed to determine factors associated with amputation for relapsed disease, as well as factors associated with a greater likelihood of wound complications. Logistic regression analysis also was conducted to determine factors associated with loco-regional recurrence. SA patients who were treated primarily with amputation were excluded from the analysis of LR-RFS and wound complications because of potential imbalances among this sub-group compared to the majority of patients treated with limb-preservation intent.

## Results

### Patient population

A total of 112 Stage II and III extremity STS cases were identified. Table 1 lists patient demographics. The median follow-up was 22.1 months (range 2.5 to 96.4 months). For SA, median follow-up was 26.6 months (range = 2.5 to 96.4 months); for NCR, 18.4 months (range = 4.5 to 95.3 months); and for NR, 29.4 months (range = 3.0 to 90.9 months). A majority of patients (79%) had lower extremity involvement, but there were no significant differences observed between disease site and treatment type. The median tumor size for the cohort was 7.9 cm (range = 0.4 cm - 29.6 cm). The median size of SA-treated tumors was significantly smaller than NCR-treated tumors (p = 0.003), but not significantly different from NR-treated tumors (p = 0.08). Tumors greater than 5 cm were treated typically with either NCR or NR (59 of 72 tumors, 82%), whereas only 40% of tumors under 5 cm received NCR or NR (12 of 30). Patients with recurrent disease did not have a significant difference in median tumor size compared to patients with primary disease (p = 0.32).

**Table 1:** Characteristics of 112 high-grade, Stage II and III soft-tissue sarcoma cases

CHARACTERISTIC	NCR	NR	SA	P
All	39	37	36	

Sex/				
Male	19	22	23	0.39
Female	20	15	13	

Grade*				
2	3	5	8	0.08
3	17	7	10	
4	16	21	13	

Age (years)				
Median (range)	58 (17-88)	71 (32-93)	54.5 (18-86)	0.03

Anatomic site				
Upper extremity	8	7	9	0.81
Lower extremity	31	30	27	

Histology				
Leiomyosarcoma	3	3	4	
Liposarcoma	4	10	4	
MFH	4	9	10	0.09
Myxofibrosarcoma	12	8	2	
Sarcoma NOS	5	2	1	
Other	11^a^	5^b^	15^c^	

Tumor size (cm)^d^				
Median (range)	10.6 (0.9-29.6)	8 (2.7-25)	4 (0.4-25)	0.01
<5 cm	4	9	19	0.0002
5-10 cm	14	13	6	
>10 cm	20	11	6	

Primary disease	37	31	23	0.002
Relapsed disease	2	6	13	

NCR, neoadjuvant chemoradiation; NR, neoadjuvant radiation alone; SA, surgery alone; MFH, malignant fibrous histiocytoma; NOS, not otherwise specified; cm, centimeters.

*: Grade data missing on 3 NCR, 4 NR and 5 SA-treated patients.

^a: ^synovial sarcoma (n = 5); epithelioid sarcoma (n = 2); myxoid liposarcoma (n = 1); malignant peripheral nerve sheath tumor (n = 1); extraskeletal myxoid chondrosarcoma (n = 1); sclerosing epithelioid fibrosarcoma (n = 1).

^b: ^synovial sarcoma (n = 1); myxoid liposarcoma (n = 2); malignant peripheral nerve sheath tumor (n = 1); clear cell sarcoma of soft tissue (n = 1).

^c: ^synovial sarcoma (n = 4); epithelioid hemangiosarcoma (n = 1); epithelioid sarcoma (n = 1); myxoid liposarcoma (n = 1); malignant peripheral nerve sheath tumor (n = 2); mixed histologies (n = 1); clear cell sarcoma of soft tissue (n = 2); adult fibrosarcoma (n = 1); mesenchymal chondrosarcoma (n = 1); angiosarcoma (n = 1).

^d: ^Does not total 112 due to missing tumor size data for 10 patients.

### Treatment

Treatments included: SA, 36 patients; NCR, 39 patients; and NR, 37 patients. One patient each in the NCR and NR group did not undergo surgery, due to the discovery of distant metastatic disease prior to surgery. NCR and NR use increased significantly after 2004, with 87% and 57% of NCR- and NR-treated patients having received therapy after 2004, respectively, compared to 69% of SA-treated patients who were treated prior to 2004 (p < 0.001). Patients with an anticipated marginal resection were selected for pre-operative therapy. Chemotherapy was utilized in a subset of these patients based on a multidisciplinary assessment of the tumor status, planned surgical procedure, co-morbidities, and performance status. When eligible, patients were enrolled on prospective trials using NCR. NCR strategies included sequential doxorubicin and ifosfamide followed by radiation (n = 1); sequential MAID followed by radiation (n = 1); sequential MAID followed by weekly cisplatin with radiation (n = 3); ifosfamide, mitomycin, doxorubicin and cisplatin with radiation (n = 7); gemcitabine plus docetaxel with radiation (n = 1); mitomycin, doxorubicin and cisplatin (without ifosfamide) with radiation (n = 1). A regimen of cisplatin weekly with radiation (n = 20) was typically used as the NCR regimen for patients treated off-protocol. This regimen was selected for its radiosensitization properties, for its limited acute toxicity, and its relative ease of standardization. No chemotherapy-related information was available for 5 NCR-treated patients because they received chemotherapy elsewhere and returned to MCA for surgery only.

The median external beam irradiation (EBRT) dose was 50.4 Gy in 28 fractions (range 25.2 Gy in 14 fractions to 54 Gy in 30 fractions). All patients were treated on linear accelerators with photon beam energies between 6-18MV using standard once-daily fractionation sizes of 1.8-2.0 Gy. Most of the patients (n = 58) were treated using three-dimensional conformal radiation techniques, but, more recently, intensity modulated radiation therapy (IMRT) was used for selected patients (n = 10). Details of radiation therapy planning were not available for 8 patients treated at outside facilities. No significant differences in the use of IOERT versus perioperative brachytherapy were observed between the NCR and NR groups; no SA patients received IOERT or perioperative brachytherapy. There were no significant differences in use of IOERT or brachytherapy with regard to patient age or sex. No significant difference in median tumor size could be detected between IOERT and perioperative brachytherapy groups (p = 0.52).

### Surgical outcome

Among patients undergoing limb preservation surgery, R0 resections were achieved in 81 patients (88%). R1 resections occurred in 11 patients (12%). As noted, 2 patients did not undergo resection due to discovery of distant metastatic disease prior to surgery. In the limb preservation group, R0 resections were achieved in 91%, 86% and 86% of NCR, NR, and SA-treated patients, respectively. As shown in Table 2, no significant differences in R0 resection rate could be detected between NR and SA (p = 0.95), NCR and SA (p = 0.55), or NCR and NR (p = 0.45). Periosteal or nerve stripping was performed in 25 patients undergoing limb preservation surgery (SA, 2 patients; NCR, 17 patients; NR, 6 patients). Patients treated with NCR or NR were significantly more likely to have periosteal or nerve stripping performed compared to SA-treated patients (p = 0.01).

**Table 2:** Outcomes of surgical resections among 92 high-grade, Stage II and III soft-tissue sarcoma cases treated with limb preservation

RESECTION TYPE	NCR	NR	SA
R0	32	30	19

R1	3	5	3

p = 0.55 NCR-SA; p = 0.45 NCR-NR; p = 0.95 NR-SA

NCR, neoadjuvant chemoradiation; NR, neoadjuvant radiation alone; SA, surgery alone; R0, surgical resection with microscopically negative margins; R1, surgical resection with margins involved microscopically.

Of the 112 patients analyzed, 18 patients had a limb amputation (16%). The median tumor size among these patients was 6.1 cm (range 0.8-18.5 cm) compared to 7.9 cm (range 0.4-29.6 cm) among patients with limb preservation (p = 0.45). Among SA-treated patients, 14 patients (39%) had a limb amputation, 6 of whom had tumors larger than 5 cm. Limb amputation occurred in 3 NCR-treated patients (8%), all with tumors larger than 5 cm. In the NR group, 1 patient (3%) had a limb amputation, with a tumor of 5.5 cm. There was no significant difference in the limb amputation rate between NCR-treated and NR-treated patients (p = 0.32). Patients presenting with recurrent disease were significantly more likely to have limb amputation than patients with primary disease (43 vs. 10%; p = 0.001). Among patients treated for recurrent disease, all limb amputations occurred in the SA group compared to no amputations for patients treated with NCR or NR (p = 0.002). Logistic regression analysis of patients undergoing amputation for recurrent disease showed that these patients were not more likely to have received prior chemotherapy or radiation than patients with recurrent disease receiving limb preservation (p = 0.77).

### Local Recurrence

**Figure 1:**
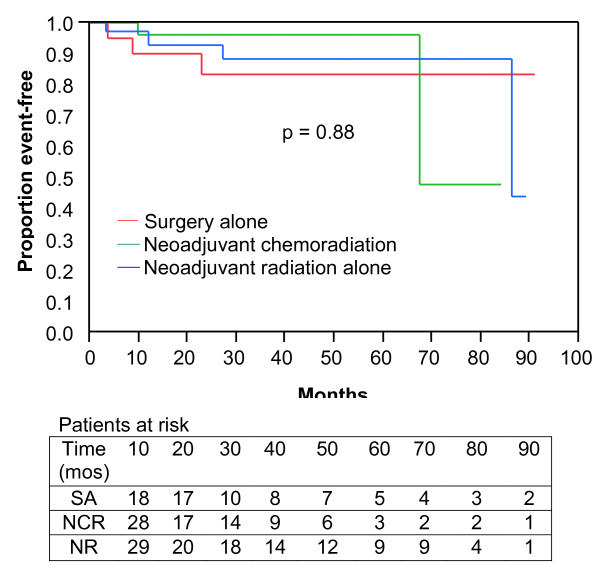
**Loco-regional relapse free survival**. Kaplan-Meier plot of 92 Stage II and III extremity soft-tissue sarcoma patients treated with limb-preservation by treatment modality (surgery alone, neoadjuvant chemoradiation, or neoadjuvant radiation alone).

Among patients treated with limb-preservation intent, loco-regional recurrences occurred in 12 patients, 4 in each treatment group. At 3 years, freedom from local recurrence was 84%, 88%, and 96% for SA, NR, and NCR respectively (Figure 1; p = 0.88). Logistic regression analysis of factors associated with loco-regional recurrence found no association between age at diagnosis (p = 0.72) or tumor site (upper extremity vs. lower extremity; p = 0.2) and recurrence risk. Patients presenting with recurrent disease (OR 11.6; p = 0.01) and tumor size greater than 5 cm (OR 9.4; p = 0.01) were more likely to have a loco-regional recurrence on logistic regression analysis. None of the ten patients treated with IMRT have developed a local recurrence, but any possible differences in local control based on radiation technique did not reach statistical significance (p = 0.43).

### Distant Metastases

**Figure 2:**
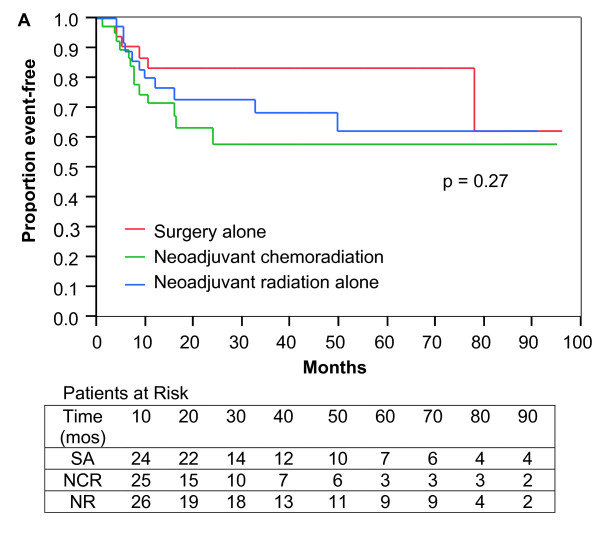
**Distant metastasis free survival**. Kaplan-Meier plot of 112 Stage II and III extremity soft-tissue sarcoma patients treated with surgery alone, neoadjuvant chemoradiation, or neoadjuvant radiation alone.

**Figure 3:**
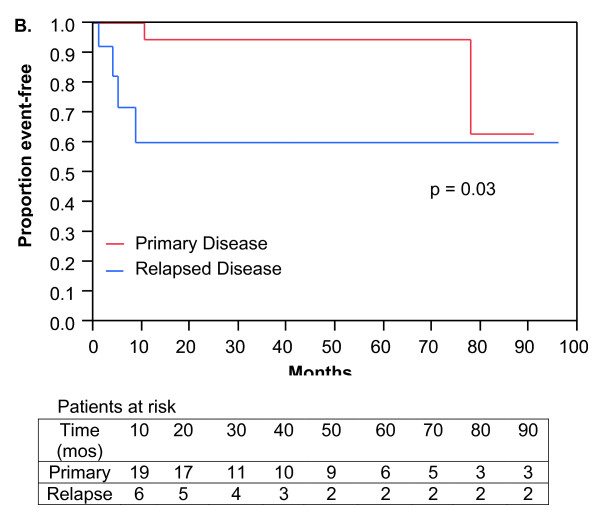
**Distant metastasis free survival**. Kaplan-Meier plot of 36 patients treated with surgery alone for primary versus relapsed disease.

Metastatic disease developed in 30 patients. Three-year DMFS was 83%, 68%, and 58% for patients treated with SA, NR, and NCR, respectively, but these were not statistically significant differences (Figure 2; p = 0.27). DMFS was significantly inferior at 3 years for patients treated with SA for recurrent disease (60%) compared to patients treated with SA for primary disease (94%; Figure 3; p = 0.03). In contrast, no differences in DMFS for patients with relapsed or primary disease treated with NCR or NR could be found.

### Overall survival

**Figure 4:**
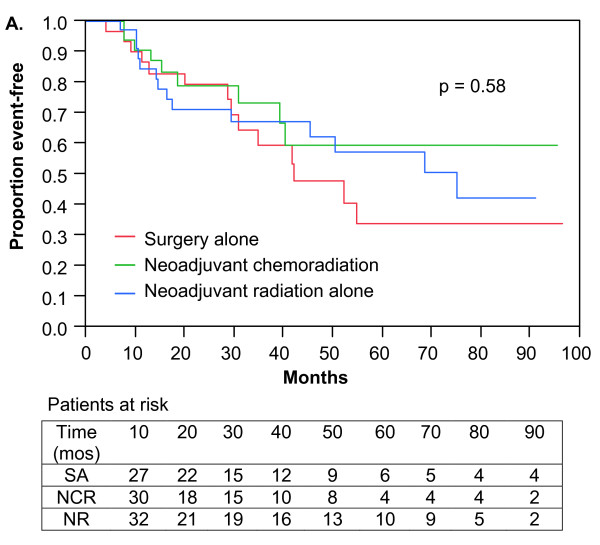
**Overall survival**. Kaplan-Meier plot of 112 Stage II and III extremity soft-tissue sarcoma patients treated with surgery alone, neoadjuvant chemoradiation, or neoadjuvant radiation alone.

**Figure 5:**
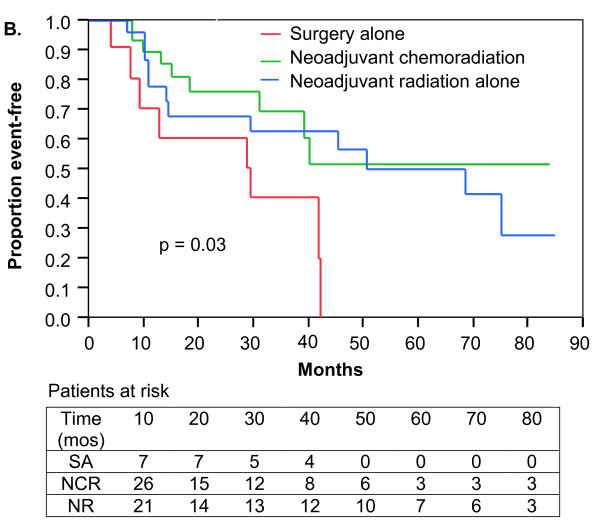
**Overall survival**. Kaplan-Meier plot of 70 patients with tumors greater than 5 cm treated with surgery alone, neoadjuvant chemoradiation, or neoadjuvant radiation alone.

The median OS was 54.7 months (95% CI; range 41.6 to 96.4 months). No significant differences in OS were observed among the treatment groups (Figure 4). Three-year OS was 59%, 67%, and 73% for SA, NR, and NCR, respectively (p = 0.58). For patients with tumors greater than 5 cm, superior OS was observed for patients treated with NCR versus SA (3-year OS 69 vs. 40%; p = 0.03; Figure 5). OS also appeared improved for patients with tumors greater than 5 cm treated with NR versus SA (3-year OS 63 vs. 40%; p = 0.02; Figure 5). There was no difference in OS among patients with tumors greater than 5 cm treated with NCR compared to NR (p = 0.57). Table 3 summarizes the LR-RFS, DMFS, OS, and limb preservation rates by treatment modality, with an additional summary of these outcomes by primary or recurrent disease status.

**Table 3:** Treatment outcomes with regard to overall survival, disease relapse (local, distant) and limb preservation by treatment method and disease presentation among 112 Stage II/III extremity soft-tissue sarcoma cases

Treatment/Disease Presentation	**No**. Pts	Survival Median (mos)	Overall Survival (%)			Local recurrence (%)			Distant Metastases (%)			Limb Preserved	
			3-yr	5-yr	P	No (%)	3-yr	p	No. (%)	3-yr	P	No. (%)	P
**SA**	36	41.9	59	34		4 (11)	84		6 (17)	83		22 (61)	

Primary	23	51.9	68	35	0.23	1 (4)	95	0.02	2 (9)	94	0.03	18 (78)	0.005

Recurrent	13	24.3	39	39		3 (23)	47		4 (31)	60		4 (31)	

**NR**	37	74.4	67	57		4 (11)	88		11 (30)	68		1 (3)	

Primary	31	74.3	67	61	0.75	2 (6)	94	0.006	9 (29)	71	0.63	29 (94)	0.54

Recurrent	6	37.4	67	*		2 (33)	56		2 (33)	50		6 (100)	

**NCR^¶^**	39	*	73	59		4 (10)	85		13 (34)	58		34 (87)	

SA, surgery alone; NR, neoadjuvant radiation alone; NCR - neoadjuvant chemoradiation; *, not reached; ¶, only 2 patients in NCR group had recurrent disease and have not developed local recurrence, distant metastases and were living at time of analysis.

### Toxicity and wound complications

Any-toxicity recorded was significantly higher among NCR-treated patients (21 of 39 patients, 54%) compared to NR-treated patients (10 of 37 patients, 27%; p = 0.02). No toxicity was documented among SA-treated patients, significantly less when compared to toxicity among NCR-treated patients (p < 0.0001). The most common toxicity among NCR-treated patients was wet desquamation in the EBRT field and gastrointestinal toxicity (nausea) from chemotherapy, each in 5 patients. Wet desquamation occurred in 4 patients treated with NR. Other toxicities observed in NCR-treated patients included myelosuppression (n = 2), electrolyte imbalance (n = 1), elevated liver biochemistries (n = 1), ifosfamide-related encephalopathy (n = 1), and venous thromboembolism (n = 1). No long term complications were documented.

Wound complications occurred in 19 of 38 (50%) NCR-treated patients (1 had limb amputation), 15 of 36 (42%) NR-treated patients, and 4 of 36 (11%) SA-treated patients (1 had limb amputation). Excluding patients treated with limb amputation, the rate of wound complications was significantly higher among the NCR-treated group compared to SA (p = 0.003; Table 4). It also was higher among the NR-treated group compared to SA (p = 0.02). Wound complication rates were not significantly different between NR and NCR groups for patients treated with limb preservation (p = 0.36). The majority of wound complications occurred among lower extremity tumors in each group (34 of 38 total wound complications). Significantly more limb-preservation patients who were treated with NCR and IOERT/perioperative brachytherapy had wound complications (16 of 30 patients, 53%) compared to NR-treated patients treated with IOERT/perioperative brachytherapy (11 of 25 patients, 44%, p = 0.009). However, using logistic regression analysis, no significant associations were found between the incidence of wound complications and the use of NCR or NR (OR 3.39; p = 0.21), use of IOERT or perioperative brachytherapy (OR 4.61; p = 0.21), or tumor size (OR 1.06; p = 0.37; Table 5).

**Table 4:** Wound complications among 92 high-grade, Stage II and III soft-tissue sarcoma cases treated with limb preservation

TREATMENT TYPE	NO. OF WOUND COMPLICATIONS	SITE
NCR	18	16 LE, 2 UE

NR	15	13 LE, 2 UE

SA	3	3 LE, 0 UE

p = 0.003 NCR-SA wound complications; p = 0.02 NR-SA wound complications; p = 0.36 NCR-NR wound complications;

NCR, neoadjuvant chemoradiation; NR, neoadjuvant radiation alone; SA, surgery alone; LE, lower extremity; UE, upper extremity.

**Table 5:** Logistic regression analysis of factors associated with wound complications

Variable	OR	p value
Age at diagnosis	1.00	0.8

Tumor size (cm)	1.06	0.31

Sex (male versus female)	1.12	0.82

Use of IOERT/Brachytherapy	4.61	0.21

Use of NCR or NR versus SA	3.39	0.21

Upper extremity versus lower extremity	0.47	0.33

IOERT, intra-operative electron radiation therapy; NCR, neoadjuvant chemoradiation; NR, neoadjuvant radiation alone; SA, surgery alone.

## Discussion

The primary treatment for Stage II and III extremity STS is typically surgery combined with pre- or post-operative radiation. Chemotherapy remains a controversial component of management. Based on the results of this study, NCR does not appear to improve outcomes compared to NR.

Neither NCR nor NR appeared to improve LR-RFS compared to SA. Previous phase III randomized trials have shown pre- and post-operative EBRT [14-19] and peri-operative brachytherapy [20-22] improve LR-RFS compared to SA. Our findings are likely impacted by the high degree of pre-treatment patient selection. Factors such as tumor grade, large size, and location relative to neuro-vascular structures or bone typically prompt referral for multimodality pre-operative therapy. Accordingly, given that patients in the NCR and NR cohorts had significantly larger tumor sizes and were more likely to undergo periosteal or nerve stripping, the equivalent local control likely reflects the benefit of neoadjuvant therapy to SA, but also lessens the likelihood of finding a significant improvement in local control with neoadjuvant treatment. No patients treated with IMRT experienced loco-regional recurrence, but no definitive conclusions can be made with regards to radiation technique and local failure. IMRT has previously been demonstrated to result in equivalent or possibly superior local control compared to conventional radiation planning [23].

NCR did not improve the R0 resection rate compared to NR or SA. This finding is similar to a randomized trial of NR followed by surgery versus surgery with post-operative radiation [15]. In that study, negative microscopic margins were seen in 83% of patients treated with NR and 85% of patients treated with post-operative radiation, suggesting no difference in surgical outcome with either strategy [15]. Therefore, as in previous studies, we are unable to demonstrate an improvement in surgical outcomes with pre-operative therapy.

No improvement in DMFS or OS was detected with NCR compared to SA or NR. Due to the heterogeneity of chemotherapy regimens used in this study cohort, we are unable to determine which, if any, chemotherapy regimen added to pre-operative radiation is optimal for impacting DMFS. Additionally, we cannot conclude which, if any, chemotherapy regimen added to pre-operative radiation might impact OS. The 5-year OS with NCR we found initially appears inferior to other studies of NCR and NR, in which 5-year OS up to 90% has been reported [8,24]. However, one analysis reported OS of 66% at 5 years for patients with tumors measuring 6-10 cm [24]. Therefore, the apparently inferior OS we observed with NCR compared to other studies likely is due to selection of higher risk patients with a larger median tumor size in our cohort. As in previous studies, addition of radiation to surgery does not appear to impact OS compared to SA [14,20,25].

We are unable to conclude whether pre-operative treatment with either NCR or NR improves limb preservation rate. A higher rate of limb amputations among SA-treated patients was observed compared to the NCR and NR groups. However, most of these SA-treated patients were deemed poor limb preservation candidates at presentation. Therefore, conclusions cannot be made as to whether a neoadjuvant strategy improved limb preservation. Differences in limb preservation rates between NCR and NR were not detected, making it unclear if the addition of chemotherapy to pre-operative therapy improves limb preservation outcomes. Logistic regression analysis showed that patients with recurrent disease treated with limb amputation were not more likely to have received previous chemotherapy or radiation than patients undergoing limb preservation for recurrent disease. Thus, many relapsed patients treated with SA possibly could have received NCR or NR, but it is likely that their disease presentation itself precluded functional limb-preservation.

A possible advantage of pre-operative treatment is the improvement in OS observed among patients with extremity STS larger than 5 cm. When compared to SA, OS was improved significantly both by NCR and NR in this subset of patients. However, no difference in OS was found between NCR and NR-treated patients with extremity STS larger than 5 cm, suggesting that the OS benefit may be derived mainly from pre-operative radiation therapy rather than from chemotherapy. No randomized controlled trials have compared NCR to SA, although previous studies failed to demonstrate an OS benefit when radiation was added to surgery versus SA [14,20,25]. Thus, the potential OS advantage for patients with large extremity STS treated pre-operatively, as suggested by our data, is intriguing, and should be confirmed prospectively. Caution must be used when interpreting this finding, since only 12 patients with extremity STS larger than 5 cm were treated with SA.

An inferior DMFS was observed among patients presenting with recurrent disease treated with SA compared to patients with primary disease treated with SA. This result suggests that patients presenting with recurrent extremity STS likely have micrometastases at the time of relapse. Such patients might benefit from more aggressive multi-agent chemotherapy either pre- or post-operatively. An improvement in DMFS for recurrent patients given chemotherapy could not be demonstrated in this analysis, although the exceedingly small number of relapsed patients (n = 2) treated with NCR greatly limits our ability to make conclusions about the value of chemotherapy for improving DMFS in these patients. Further analyses of outcomes among a higher number of patients with recurrent disease should be conducted to determine whether chemotherapy is beneficial in this subgroup of patients.

Potential drawbacks of NCR are increased toxicity and wound complication rates. In a phase III trial of pre- versus post-operative radiation without chemotherapy, wound complications occurred in 35% of patients treated with pre-operative radiation therapy [15]. While wound complication rates of just 7.5% have been reported with intra-arterial doxorubicin and radiation in single institution experience [13], a multi-center trial of intra-arterial doxorubicin with radiation reported a 41% wound complication rate [9]. Logistic regression analysis did not find a significant association between use of NCR or NR and wound complications, nor with use of IOERT/perioperative brachytherapy. Additionally, we found no significant difference in the wound complication rate between NCR and NR. We cannot conclude that NCR worsens the wound complications rate based on these results. The apparent higher rate of wound complications we observed may be attributable to different definitions of wound complications among studies. Due to small patient numbers, it is not entirely clear that the observed rate of wound complications in our study is significantly different than rates reported in other studies. Working closely with our plastic surgery colleagues, we have not appreciated long-term negative impacts on function or quality of life in patients who experience wound complications. Beyond wound complications, the overall degree of toxicity associated with NCR appeared higher compared to NR. However, we were unable to grade toxicities from medical records, and due to inconsistencies in documentation, the increased rate of any-toxicity with NCR reported here must be viewed with caution. Our group is actively pursuing further analyses of wound complications in order to better understand these findings and improve practice.

There are several limitations to this study. Foremost is its retrospective nature, which may lead to biased results because of potential imbalances in the treatment groups being compared. Secondly, we studied a diverse mixture of patients, with differing primary disease sites, limiting conclusions as to which primary disease location might benefit most from neoadjuvant therapy. Furthermore, any conclusion as to which chemotherapy regimen may be optimal is limited by the relatively small numbers of patients were treated over the 11-year period with various chemotherapy agents and schedules.

## Conclusions

Despite the limitations of the methodology, the results of this study have merit. We conclude that both NCR and NR result in a low rate of loco-regional relapse, high rates of limb preservation, and acceptable toxicity. The improved OS of patients with tumors greater than 5 cm treated with pre-operative therapy (both with NCR and NR) compared to patients with tumors greater than 5 cm receiving SA is compelling. We continue to track outcomes of patients treated with weekly cisplatin given with radiation, but cannot make conclusions about its effectiveness from the available data at this time. Wound complications remain an important management issue for patients treated with a pre-operative strategy, but NCR did not significantly increase the risk of wound complications compared to NR.

In addition to cure, goals of extremity STS therapy include limb preservation, minimizing treatment-related toxicity, and maximizing quality of life both during and after treatment. The results of this analysis suggest that NCR and NR appear to be effective strategies for Stage II and III STS, perhaps with improved outcomes compared to SA, but NCR is not clearly superior to NR.

## List of Abbreviations

STS: soft tissue sarcoma; NCR: neoadjuvant chemoradiation; MAID: mesna, doxorubicin, ifosfamide and dacarbazine; IMAP/MAP: ifosfamide, mitomycin, doxorubicin and cisplatin; MCA: Mayo Clinic in Arizona; NR: neoadjuvant radiation; SA: surgery alone; MRI: magnetic resonance imaging; AJCC: American Joint Committee on Cancer; IOERT: intra-operative electron radiation therapy; OS: overall survival; LR-RFS: loco-regional recurrence-free survival; DMFS: distant metastases-free survival; EBRT: external beam irradiation; IMRT: intensity modulated radiation therapy.

## Competing interests

The authors declare that they have no competing interests.

## Authors' contributions

All authors have read and approved the final manuscript.

KKC was involved in clinical care of patients included in the data set, conceived of the study, collected and analyzed data, and helped draft the manuscript.

JBA was involved in clinical care of patients included in the data set, conceived of the study, collected and analyzed data, and helped draft the manuscript.

CPB was involved in clinical care of patients included in the data set and reviewed the manuscript.

AJS was involved in clinical care of patients included in the data set and helped draft the manuscript.

MDC was involved in clinical care of patients included in the data set and helped draft the manuscript.

ACD assisted with statistical analysis of the data.

LLG was involved in clinical care of patients included in the data set and helped draft the manuscript.

TRF was involved in clinical care of patients included in the data set and reviewed the manuscript.
